# Vascular Occlusions following Ocular Surgical Procedures: A Clinical Observation of Vascular Complications after Ocular Surgery

**DOI:** 10.1155/2017/9120892

**Published:** 2017-07-11

**Authors:** Charlotte Fischer, Anne Bruggemann, Annette Hager, Josep Callizo Planas, Johann Roider, Hans Hoerauf

**Affiliations:** ^1^University Eye Clinic, Georg-August-Universität Göttingen, Göttingen, Germany; ^2^Eye Clinic, University Medical Center Schleswig-Holstein, Lübeck, Germany; ^3^University Eye Clinic, Charité, Berlin, Germany; ^4^Eye Clinic, University Medical Center Schleswig-Holstein, Kiel, Germany

## Abstract

**Background:**

Ocular vascular occlusions following intraocular procedures are a rare complication. We report a case series of patients with retinal vascular occlusions or anterior ischemic optic neuropathy (AION) after anterior and posterior segment surgery and demonstrate possible risk factors.

**Methods:**

Observational case series.

**Results:**

In ten patients, vascular occlusions were observed within ten weeks after intraocular surgery: branch retinal arterial occlusion (BRAO) (*n* = 2), central retinal artery occlusion (CRAO) (*n* = 2), central retinal vein occlusion (CRVO) (*n* = 1), branch retinal vein occlusion (BRVO) (*n* = 1), anterior ischemic optic neuropathy (AION) (*n* = 3), and combined central artery and vein occlusion (*n* = 1). AION occurred later (27–69 d) than arterial occlusions (14–60 d) or venous occlusions (1-2 d). In all cases, either specific surgical manipulations or general vascular disorders were identified as risk factors. In addition to general cardiovascular risk factors (arterial hypertension *n* = 6, diabetes mellitus *n* = 4), internal workup disclosed bilateral stenosis of the carotid arteries (*n* = 1) and myeloproliferative syndrome (*n* = 1).

**Conclusion:**

Vascular occlusions after surgical ocular procedures seem to be more frequent when cardiovascular diseases coexist. Surgical maneuvers and intra- or postoperative pressure changes may act as a triggering mechanism in patients with underlying systemic cardiovascular disorders. Affected patients should undergo thorough internal examination to identify possible underlying diseases.

## 1. Introduction

While visual loss due to central retinal artery occlusion (CRAO) or branch retinal arterial occlusion (BRAO) following gas endotamponade and intraocular pressure (IOP) rise is a well-recognized complication [1, 2], cases of central retinal venous occlusion (CRVO) or branch retinal venous occlusion (BRVO) after vitreoretinal surgery have not been reported so far. And although it has been shown that ocular blood flow decreases significantly following scleral buckling procedures [3, 4], just one report exists on postoperative retinal arterial occlusion after retinal detachment surgery [5].

However, nonarteritic anterior ischemic optic neuropathy (AION) and retinal arterial occlusions are a more common event after routine phacoemulsification with retro- or peribulbar anesthesia. Even delayed occurrences up to six months postoperatively have been described [6].

Intraocular surgical procedures and especially vitreoretinal procedures frequently combine different surgical maneuvers. Thus, after the occurrence of postoperative vascular occlusion, the causative factor is hard to identify.

In this observational case series, we present ten patients with vascular occlusions in the posterior segment following different vitreoretinal or intraocular procedures without retro- or peribulbar anesthesia and try to identify possible underlying risk factors which may have triggered or caused these occlusions.

## 2. Methods

In observational series of ten patients who underwent intraocular procedures, vascular occlusion occurred within ten weeks postoperatively. The patients underwent intraocular surgery between 1995 and 2015. Demographic dates, indication/diagnosis, surgical procedures, type and occurrence of vascular occlusion, and visual results are presented in Table 1. Research adhered to the tenets of the Declaration of Helsinki.

All patients underwent a complete ophthalmologic examination and an extensive workup by an internal physician to identify possible coexisting cardiovascular risk factors.

### 2.1. Demographic Data and Ophthalmic History

Of the ten patients, four were male and six were female. The mean age was 68.4 years (ranging from 53 to 79 years). They underwent different intraocular and vitreoretinal procedures due to rhegmatogenous retinal detachment (*n* = 4), tractional retinal detachment (*n* = 1), macular hole (*n* = 2), and senile cataract (*n* = 3). None of the patients have had previous intraocular surgery in the affected eye. All patients were operated in general anesthesia. No patient received a retro- or peribulbar injection. Further ocular diseases included a chronic open-angle glaucoma in one patient. A routine postoperative antiembolic treatment prophylaxis was not administered to the patients.

### 2.2. Procedures

Vitreoretinal procedures included pars plana vitrectomy (*n* = 5), conventional scleral buckling (meridional sponge) (*n* = 2), and uneventful extracapsular cataract extraction with phacoemulsification and implantation of a posterior chamber lens (*n* = 3). In three patients who underwent vitrectomy, an additional encircling band was applied due to multiple peripheral retinal breaks, and in four of the vitrectomized eyes, membrane peeling was performed.

## 3. Results

### 3.1. Vascular Occlusions

During the postoperative course, the following retinal vascular occlusions were identified: BRAO (*n* = 2), CRAO (*n* = 2), BRVO (*n* = 1), CRVO (*n* = 1), AION (*n* = 3), and combined central artery and vein occlusion (*n* = 1). Whereas retinal venous occlusions were observed within the early postoperative period (1-2 d), the arterial occlusions varied between postoperative days 14, 19, 23, and 60 (median 21 d), respectively. AION occurred even later (27 d, 65 d, and 69 d). However, the diagnosis may be delayed by gas endotamponade after vitrectomy. Still, central retinal artery occlusion with no light perception would have been noticed during the routine examination at the first postoperative day, and hand motion was documented on postoperative day two for patient numbers 1, 2, 6, and 10.

### 3.2. Visual Acuity and Intraocular Pressure

Preoperative best-corrected visual acuity (BCVA) of all patients ranged between hand motion (logMAR 2.0) and 20/25 (logMAR 0.1). Postoperative BCVA depended on the underlying disease, the type of postoperative vascular occlusion, and macular involvement. As a consequence of the vascular occlusion, BCVA worsened in six of 9 patients.

IOP was elevated postoperatively in three of eight eyes with moderate peaks (30 and 32 mmHg), respectively.

### 3.3. Preexisting Cardiovascular Risk Factors and Results of Postoperative Internal Workup

In six patients, preexisting cardiovascular risk factors were known: chronic arterial hypertension (*n* = 6), cardiac arrhythmia (*n* = 3), and diabetes mellitus type II (*n* = 4). Postoperative internal workup revealed additional cardiovascular risk factors in two patients. In patient number 2, a bilateral severe stenosis of the inner carotid artery (ACI) was detected. In this patient, an ACI bypassing was scheduled immediately. In patient number 5, a myeloproliferative syndrome was diagnosed. In all patients with arterial occlusion, an arteritic cause was ruled out. Patient number 3 had a bilateral Terson syndrome due to subarachnoid hemorrhage, but no further cardiovascular risk factors were diagnosed.

Only in one of the ten patients, no comorbidity could be identified.

## 4. Discussion: Possible Triggering Mechanisms

### 4.1. Risk Factor: Anesthesia

Retro- or peribulbar anesthesia may cause a direct iatrogenic injury of the nerve or a retrobulbar hemorrhage resulting in compression of the optic nerve. Also, vasoconstriction by an anesthetic agent depleting blood flow or triggering a stress-induced vascular spasm has been reported [7, 8]. No patient in our series underwent a retro- or peribulbar anesthesia. In all patients, surgery was performed in general anesthesia. Iatrogenic retinal arterial occlusion was reported following vitreoretinal surgery with gas endotamponade and the use of nitrous oxide during general anesthesia leading to expansion of intraocular gas and consecutive severe IOP rise [9]. In the reported patients, no nitrous oxide was used and since 2003, only modern short-term acting anesthetic agents were used. IOP rise and a decrease in ocular blood flow were also described after tracheal intubation and interpreted as a stress response associated with intubation [10]. In the listed patients who underwent intraocular surgery in the late nineties tracheal intubation, since 2003, larynx masks were used. Marked effects on ocular blood flow may be further associated with severe changes in blood volume and blood pressure. During the procedures, there was no loss of blood and blood pressure was well controlled. However, a well-controlled blood pressure during general anesthesia in an otherwise hypertensive patient may have caused reduced ocular perfusion. This issue is also discussed in the next chapter.

### 4.2. Risk Factor: Elevation of IOP

Severe IOP elevation after vitreoretinal surgery with gas endotamponade was reported in 20 up to 60% of patients [1, 2]. The endotamponade may lead to a forward displacement of the lens-iris diaphragm resulting in anterior chamber flattening and secondary angle-closure glaucoma [11]. The risk is increasing when expandible gas mixtures are used depending on the concentration and type of gas. Due to the capacity of gas expansion and its time course, the highest rates of IOP elevation should be expected approximately 24 to 48 hours postoperatively [12]. Thompson et al. reported peaks of pressure also between day 2 and 2 weeks after macular hole surgery [13]. The second reason for anterior chamber flattening is ciliary body edema and choroidal swelling caused by extensive laser or cryocoagulation, scleral buckling, or choroidal hemorrhage [14]. These mechanisms may have played a role in patient numbers 1, 2, 5, and 10. Three of them showed a moderate postoperative IOP elevation (peaks of 30 and 32 mmHg) during early postoperative period (days 1 and 2) and normal IOP when discharged. In the early postoperative period, no loss of light perceptions was documented in any of our patients in contrast to other reports [15]. The IOP was not measured in the first hours after surgery since routinely 500 mg of intravenous acetazolamide after intravitreal procedures using gas or buckling procedures was administered.

**Figure 1:**
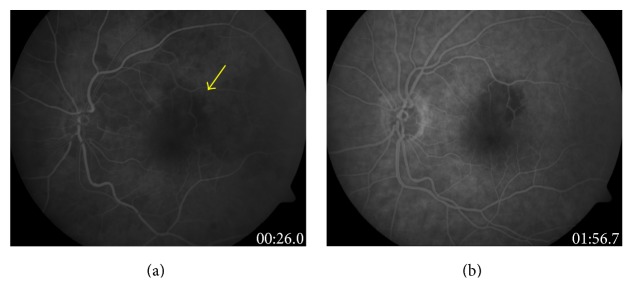
Fluorescein angiography of patient number 6 (BRAO) two weeks after successful macular hole surgery (a) demonstrating a small macular arterial occlusion at the superior arcade (arrow) and (b) showing the nonperfused retinal area.

**Figure 2:**
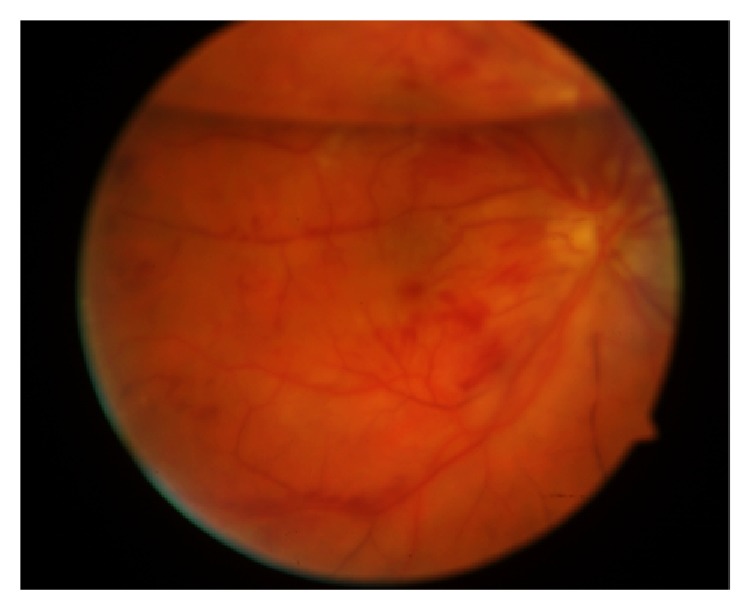
Fundus photography of patient number 10 showing a C_2_F_6_ 40% gas-filled vitreous cavity with combined central artery and vein occlusion after primary vitrectomy and encircling band for rhegmatogenous retinal detachment.

In patient number 1, SF_6_ 40% gas was used, which is an expandible mixture and therefore a possible risk factor for IOP elevation and arterial occlusion, though it was an incomplete tamponade, and the maximum IOP measured on day two was moderately increased (30 mmHg). Furthermore, the vascular occlusion presented on day 23 where IOP was already normalized. In patient number 2, C_2_F_6_ 10% gas was used; in patient number 6, C_2_F_6_ 15%; and in patient number 10, C_2_F_6_ 14%, while these are nonexpandible mixtures. In all these four cases with gas endotamponade, patients presented with retinal arterial occlusion 14, 19, 23, and 32 days after surgery when intraocular gas had almost been reabsorbed. In patient number 1 and number 6, BRAO was diagnosed (Figure 1); in patient number 2, CRAO; and in patient number 10, combined central artery and vein occlusion (Figure 2).

In cases of AION after cataract extraction, the combination of an increased intraocular pressure postoperatively and a drop in systemic blood pressure due to the anesthesia was discussed as a causative mechanism especially in early-period events [16]. However, the influence of intraoperative IOP fluctuations during phacoemulsification or vitrectomy is dependent on the applied aspiration pressure [17, 18]. The higher the aspiration pressure, the higher the IOP up to 85 mmHg.

### 4.3. Risk Factor: Perfusion

Ischemia syndromes of the anterior segment after scleral buckling procedures are a severe but extremely rare vascular complication. However, despite being not clinically evident, it has been shown that retinal and choroidal blood flow is frequently compromised and the episcleral vein pressure is elevated [3, 4]. Even though ocular blood flow might be decreased after scleral buckling procedures, patients usually do not develop retinal vascular occlusion following buckling surgery. In patient number 4 and patient number 5 who underwent a conventional scleral buckling procedure either with subretinal drainage (number 4) or with intraoperative paracentesis (number 5) and cryopexy, neither marked postoperative changes of IOP nor significant flattening of the anterior chamber was noted. On postoperative day one, ophthalmoscopy in both cases showed a reattached retina; no vascular changes were documented. However, on postoperative day two in patient number 5 and on day 27 in patient number 4, nonischemic CRVO and AION occurred with a visual decrease, respectively. Changes in ocular blood flow and postoperative blood pressure might have played a role in developing venous stasis and occlusion. Patient numbers 1 and 2 who underwent pars plana vitrectomy and gas endotamponade were treated with an additional encircling band, which as well might have triggered their vascular occlusions.

### 4.4. Risk Factor: Direct Vessel Injury

A visual decrease after panretinal photocoagulation for proliferative retinopathy has been reported in the literature usually due to the development of cystoid macular edema [19]. However, Kangas et al. described five out of seven patients who suffered a visual loss that could not be explained by any observable ocular complication of the procedure such as cystoid macular edema, and all patients recovered vision [15]. The induction of arterial occlusion by a laser lesion alone with regular exposure time seems unlikely; however, it has been shown, based on a severely damaged ischemic vascular system and an impaired autoregulation of the retinal blood flow, that a normal laser lesion may be sufficient to cause arterial occlusion, such as in compromised retinal vessels of diabetic patients [20].

Patient number 3 showed BRVO close to the location of intraretinal mechanical manipulation including membrane peeling. Additionally, endolaser photocoagulation was performed. A direct vascular trauma cannot be ruled out but was not documented in the surgical report.

### 4.5. Risk Factor: Systemic Diseases

Known risk factors for retinal arterial occlusions are arterial hypertension, cardiac arrhythmia, arteriosclerosis, hypercholesterinemia, diabetes, and rare blood diseases [21]. Patient numbers 1, 4, 6, 7, 8, and 9 suffered from hypertension; patient numbers 4, 7, 8, and 9 suffered from controlled diabetes mellitus; and patient numbers 1, 7, and 8 additionally suffered from cardiac arrhythmia. The most common systemic risk factors for retinal venous occlusions are age, arterial hypertension, hyperlipidemia, arterial sclerosis, and diabetes mellitus [22] and for AION are age, arterial hypertension, diabetes mellitus, hypercholesterolemia, tobacco use, systemic atherosclerosis, obstructive sleep apnea, gastrointestinal ulcers, and ischemic heart disease [20]. Preoperatively, no systemic risk factors were known in patient numbers 2 and 5 with retinal venous occlusions and in patient number 10 with combined central artery and vein occlusion.

After the vascular occlusion was diagnosed, all our patients underwent a profound examination including laboratory testing for hemophilic disorders. In one 69-year-old patient (number 10), no further systemic cardiovascular risk factor was found. In this case, a combined pathomechanism of reduced perfusion after scleral buckling procedure and IOP fluctuations caused by the gas endotamponade has to be assumed. But in two patients, the postoperative internal examination revealed underlying risk factors: in patient number 5, an early-stage myeloproliferative syndrome which can cause blood hyperviscosity and serve as a trigger or be the main reason for the observed postoperative central retinal vein occlusion and in patient number 2, a severe bilateral stenosis of the inner carotid artery which explains the central retinal artery occlusion after macular hole surgery. In both patients, the blood pressure changes and subsequent changes in the ocular blood flow may have acted as triggering mechanisms for the delayed postoperative retinal vascular occlusion.

### 4.6. Limitations of the Study

As reported patients within this study occurred within a long period of over 20 years, changes of standard surgical procedures and of general anesthesia within these two decades might have an influence on the likelihood of vascular occlusions following intraocular procedures. However, the basic principles of phacosurgery or vitrectomy and the use of tamponades did not change.

Another limitation is the heterogeneous group of patients and the variability of surgical procedures, which makes it impossible to estimate the different risk profiles; however, the intention of the authors was to make ophthalmic surgeons aware of the general risk of retinal vascular occlusions after ocular surgical procedures in patients with coexisting cardiovascular risks.

## 5. Conclusion

Vascular ocular occlusions after intraocular surgery seem to be more frequent when cardiovascular diseases coexist. Surgical manipulations causing decreased retinal and choroidal blood flow as well as pressure changes may act as triggering mechanisms on the basis of a compromised vascular system. Affected patients should undergo a careful examination to disclose possible underlying diseases. A thorough medical anamnesis is important to identify patients with a higher risk, and informed consent should include retinal vascular occlusions and AION as complications of intraocular surgery.

## Figures and Tables

**Table 1:** 

Case number	1	2	3	4	5	6	7	8	9	10
Sex	Female	Female	Male	Male	Female	Female	Female	Female	Male	Male
Age	79	72	53	64	63	75	71	75	63	69
Indication	Vitreous hemorrhage, rhegmatogenous RD	MH with several peripheral retinal degenerations	Vitreous hemorrhage, tractional RD	Rhegmatogenous RD	Rhegmatogenous RD	MH	Senile cataract	Senile cataract	Senile cataract	Rhegmatogenous RD
Surgical procedure	PPV, encircling band, radial buckle, SF_6_ (40%)	Encircling band, PPV, C_2_F_6_ (10%)	PPV, MP, endolaser photocoagulation	Buckling procedure parallel to the limbus, cryopexy, subretinal punction	Meridional buckling procedure, cryopexy	PPV, ILM peeling, C_2_F_6_ (15%)	Phaco, PCL	Phaco, PCL	Phaco, PCL	PPV, encircling band, C_2_F_6_ (14%)
Preop BCVA (logMAR)	HM (2.0)	0.4	HM (2.0)	0.7	0.5	1.3	0.4	1.0	0.5	0.1
Final BCVA (logMAR)	HM (2.0)	HM (2.0)	NA	1.0	0.4	0.5	0.8	HM (2.0)	1.0	HM (2.0)
Max. postop IOP	30	32	NA	NA	NA	16	10	12	26	32
Type of vascular occlusion	Arterial branch occlusion	Central artery occlusion	Venous branch occlusion	Anterior ischemic optic neuropathy	Incomplete central vein occlusion	Arterial branch occlusion	Anterior ischemic optic neuropathy	Central artery occlusion	Anterior ischemic optic neuropathy	Combined central artery/vein occlusion
Day of onset	23	19	2	27	1	14	65	60	69	21
Ocular risk factor	Open-angle glaucoma	None	None	None	None	None	None	PEX	Diabetic retinopathy	None
Medical risk factors	Arterial hypertension, supraventricular tachycardia	Bilateral severe stenosis of ACI	Terson syndrome	Diabetes mellitus type II, arterial hypertension	Myeloproliferative syndrome	Arterial hypertension	Diabetes mellitus type II, arterial hypertension, arterial fibrillation	Arterial hypertension, diabetes mellitus type II, cardiac arrhythmia	Diabetes mellitus type II, arterial hypertension	None

RD: retinal detachment; MH: macular hole; PPV: pars plana vitrectomy; C_2_F_6_: C_2_F_6_ gas endotamponade; SF_6_: SF_6_ gas endotamponade; MP: membrane peeling; BCVA: best-corrected visual acuity (logMAR): HM: hand motion; ACI: inner carotid artery; Phaco: phacoemulsification; NA: not acquired; PEX: pseudoexfoliation syndrome; PCL: posterior chamber lens implantation.
